# Engineered Trehalose Permeable to Mammalian Cells

**DOI:** 10.1371/journal.pone.0130323

**Published:** 2015-06-26

**Authors:** Alireza Abazari, Labros G. Meimetis, Ghyslain Budin, Shyam Sundhar Bale, Ralph Weissleder, Mehmet Toner

**Affiliations:** 1 The Center for Engineering in Medicine, Massachusetts General Hospital, Harvard Medical School, and Shriners Hospital for Children, Boston, Massachusetts, United States of America; 2 The Center for Systems Biology, Massachusetts General Hospital and Harvard Medical School, Boston, Massachusetts, United States of America; National Cancer Institute, UNITED STATES

## Abstract

Trehalose is a naturally occurring disaccharide which is associated with extraordinary stress-tolerance capacity in certain species of unicellular and multicellular organisms. In mammalian cells, presence of intra- and extracellular trehalose has been shown to confer improved tolerance against freezing and desiccation. Since mammalian cells do not synthesize nor import trehalose, the development of novel methods for efficient intracellular delivery of trehalose has been an ongoing investigation. Herein, we studied the membrane permeability of engineered lipophilic derivatives of trehalose. Trehalose conjugated with 6 acetyl groups (trehalose hexaacetate or 6-O-Ac-Tre) demonstrated superior permeability in rat hepatocytes compared with regular trehalose, trehalose diacetate (2-O-Ac-Tre) and trehalose tetraacetate (4-O-Ac-Tre). Once in the cell, intracellular esterases hydrolyzed the 6-O-Ac-Tre molecules, releasing free trehalose into the cytoplasm. The total concentration of intracellular trehalose (plus acetylated variants) reached as high as 10 fold the extracellular concentration of 6-O-Ac-Tre, attaining concentrations suitable for applications in biopreservation. To describe this accumulation phenomenon, a diffusion-reaction model was proposed and the permeability and reaction kinetics of 6-O-Ac-Tre were determined by fitting to experimental data. Further studies suggested that the impact of the loading and the presence of intracellular trehalose on cellular viability and function were negligible. Engineering of trehalose chemical structure rather than manipulating the cell, is an innocuous, cell-friendly method for trehalose delivery, with demonstrated potential for trehalose loading in different types of cells and cell lines, and can facilitate the wide-spread application of trehalose as an intracellular protective agent in biopreservation studies.

## Introduction

Trehalose is a naturally occurring alpha-linked disaccharide formed by an α,α-1,1-glucosidic bond between two α-glucose units. Synthesized mostly by a group of organisms called anhydrobiots, which include bacteria, yeast, nematodes, rotifers, tardigrades, certain crustaceans and insects, trehalose has been shown to play a key role in various types of stress-tolerance including radiation, cold and dehydration stresses in these creatures [1,2]. Studies on the role of trehalose in stress-tolerance suggest that trehalose must be presence on both sides of the cell membrane to confer protection [3–9]. It is also proposed that trehalose contributes to the formation of a stable glassy state when dried, which prevents deleterious conformational changes in proteins and significantly impedes molecular mobility, hence reducing the rate of deteriorating biochemical reactions [10–13]. For these properties and the fact that it is generally nontoxic, trehalose is an attractive protective agent in biopreservation. It is used as a common additive in pharmaceutical formulations, contributing to the stabilization of compounds in frozen or dried states [14], and it has shown promise as a protective agent against freezing and desiccation-induced damages in some mammalian cells when delivered intracellularly [15–22].

A major barrier preventing the wide-spread application of trehalose in biopreservation is the difficulties associated with the intracellular delivery of trehalose. Mammalian cells lack the genetic information for synthesis of trehalose or the expression of trehalose transporters in their membranes. Hence, invention of novel methods for the intracellular delivery of trehalose has been an ongoing investigation. Of the handful of developed methods, osmotic shock [23], liposomal delivery [24], thermal poration [21], electroporation [25], microinjection [19,26], engineered pores [18,20,27], and genetic engineering [15,28] require external, deliberate and sometimes cumbersome manipulation of the cell. The methods involving poration of the cell membrane generally allow nonspecific transport of molecules and ions other than trehalose, which result in alteration of transmembrane ionic balance and may lead to significant cell damage. Methods involving genetic interventions for the synthesis of trehalose or trehalose transporter proteins may not be suitable for clinical application. Other methods such as utilization of P_2_X_7_ purinergic membrane pores [29] and fluid-phase endocytosis [30] use native mechanisms in mammalian cells for trehalose uptake. However, native membrane pores such as P_2_X_7_ are specific to only a few cell types, and fluid-phase endocytosis is slow, cell-type dependent and involves cumulative osmotic stress during loading. Such limitations prevent the universal application of these techniques for trehalose delivery in biopreservation studies.

In this study, we investigated an approach for intracellular delivery of trehalose based on modification of trehalose chemical structure rather than manipulating the cell. It has been observed that enhancing small molecule lipophilicity often increases the propensity to cross biological membranes [31,32]. The movement of a lipophilic compound through the plasma membrane is facilitated by the availability of the extensive hydrophobic surface area, which lowers the activation energy for the membrane passage [33]. We postulated that by substituting the hydrophilic hydroxyl groups with lipophilic acetyl groups, we would facilitate its permeability across cell membranes. This was achieved through selective acetylation of trehalose to varying degrees, providing trehalose derivatives with the appropriate properties to pass cell barriers. Conveniently these derivatives can be deacetylated in the cell by non-specific esterases to yield trehalose. Specifically, we synthesized and tested three chemically-defined trehalose derivatives and studied their membrane permeability and intracellular conversion in primary rat hepatocytes. Primary hepatocytes are important cells frequently used in high throughput drug toxicity screening and pharmacokinetic/pharmacodynamic studies as well as in tissue engineering and regenerative medicine. Our results demonstrated the uptake, conversion and accumulation of acetylated trehalose derivatives in rat hepatocytes with unprecedented efficiency. For the best-performing trehalose derivative, we proposed a diffusion-reaction model to describe its membrane permeability and conversion kinetics. Engineering of trehalose chemical structure rather than manipulating the cell, is an innocuous, cell-friendly method for trehalose delivery, and can be applicable to many different types of cells and cell lines, and can facilitate the wide-spread application of trehalose as an intracellular protective agent in biopreservation studies.

## Materials and Methods

Phosphate-buffered saline tablets, HEPES buffer powder, o-phenylenediamine dihydro-chloride for rat albumin assay, thiazolyl blue tetrazolium bromide for the MTT assay, epidermal growth factor (EGF), carbonyl cyanide m-chlorophenyl hydrazone (CCCP), glucose, sucrose, glucagon and hydrocortisone were all obtained from Sigma, St. Louis, MO. Trehalose was purchased from Ferro-Pfanstiehl, Waukegan, IL, USA; trypsin-EDTA, Dulbecco’s Modified Eagle’s medium (DMEM), fetal bovine serum (FBS) were from Life Technologies, Grand Island, NY; fibronectin was obtained from BD, Franklin Lakes, NJ. The trehalose assay kit was purchased from Megazyme, Ireland and rat albumin antibody (peroxidase-conjugated sheep IgG fraction) from MPBio, Santa Ana, CA, USA.

Phosphate-buffered saline was prepared by dissolving PBS tablets in ultra-high purity (>18 mOhm) distilled water. Collagen solution was prepared following an in-house protocol from rat tail. Rat hepatocyte maintenance medium was prepared as the following: high glucose (4.5g/L), DMEM supplemented with 10% FBS, 0.02 mg/L glucagon, 20 ng/mL EGF, 7.5 μg/mL hydrocortisone and 2% (v/v) penicillin/streptomycin.

### Synthesis of trehalose derivatives

**Fig 1:**
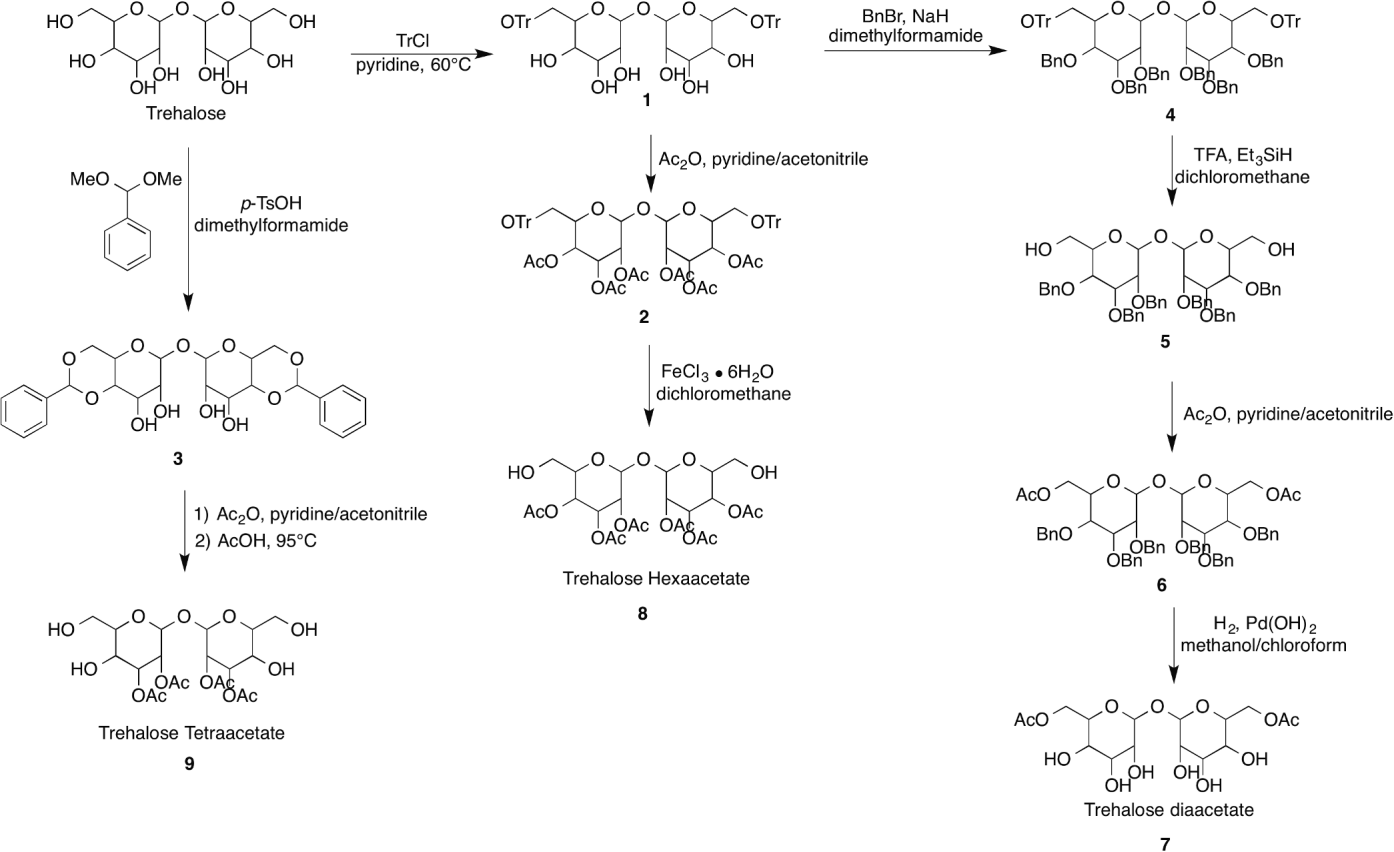
The synthesis steps for trehalose diacetate (2-O-Ac-Tre), trehalose tetraacetate (4-O-Ac-Tre) and trehalose hexaacetate (6-O-Ac-Tre).

Three trehalose derivatives were synthesized with 2, 4 and 6 degrees of acetylation as shown in Fig 1. To a solution of Trehalose in pyridine was added trityl chloride to give intermediate 1, which was subsequently acetylated with acetic anhydride to provide compound 2. Trityl deprotection of compound 2 was accomplished with Iron (III) chloride hexahydrate in methylene chloride to yield trehalose hexaacetate (compound 8). In a similar fashion trehalose tetraacetate (compound 9) was constructed by formation of the bis acetal (compound 3) from trehalose followed by acetylation and deprotection with acetic acid to afford trehalose tetraacetate (compound 9). Common synthetic intermediate 1 was used to access trehalose diaacetate Firstly, intermediate 1 was benzyl protected with benzyl bromide and sodium hydride in dimethyl formamide to give the fully protected dissacharide intermediate 4. Compound 4 was trityl deprotected with trifluoroacetic acid and subsequently acetylated with acetic anhydride to give intermediate 6. Finally, compound 6 was benzyl deprotected with palladium hydroxide in the presence of hydrogen to provide trehalose diaacetate (compound 7).

### Hepatocyte isolation

Rat hepatocytes were provided by the Cell Resource Center (CRC) at the Center for Engineering in Medicine at Massachusetts General Hospital. Briefly, hepatocytes were obtained from female Lewis rats using a two-step collagenase protocol. Two or three month old rat (Charles River Laboratories, Wilmington, MA) weighing 180–200 g were used as hepatocyte source and were maintained in accordance with National Research Council guidelines. Experimental protocols in practice at CRC for the use of hepatocytes in this study were approved by the subcommittee on Research Animal Care, Massachusetts General Hospital. Using a modification of the two-step collagenase perfusion method, which involved purification of cell suspension by means of centrifugation over Percoll, approximately 200 million hepatocytes per rat liver with 85–95% viability were routinely obtained, as evaluated by Trypan blue exclusion. Hepatocytes were kept on ice and were plated within 1 h of isolation.

### Hepatocyte culture for short-term experiments

For trehalose loading experiments, 6-well plates were treated with 25μg/mL fibronectin for 1 h prior to plating the hepatocytes, at which point the wells were washed with PBS and the hepatocytes were seeded at 1.2–1.3×10^6^ cells/well. The cells were allowed 24–48 h to attach and spread in a 10% (v/v) CO_2_ incubator at 37°C before they were incubated with acetylated variants of trehalose.

### Measurement of the uptake kinetics and deacetylation of intracellular trehalose

To understand the kinetics of acetylated trehalose permeation and conversion in rat hepatocytes, acetylated trehalose variants (2-, 4-, and 6-O-Ac-Tre) were dissolved in hepatocyte maintenance medium, and the pH of the solutions were adjusted to 7.1 using HEPES buffer (25 mM). The hepatocyte culture media were replaced with pre-warmed acetylated trehalose solution in medium, and the cells were incubated with acetylated trehalose for 1 to 12 h. The incubation concentration was 30 mM for all acetylated trehalose variants. At the end of the incubation time, the media were removed and cells were washed with 200 mM sucrose-supplemented PBS. Sucrose supplement was provided to reduce potential osmotic swelling and lysis. Then, the cells were detached from the plates by the addition of and incubation with 0.5 mL pre-warmed trypsin-EDTA solution (final concentration 0.25%) in 200 mM sucrose-supplemented PBS for 10–12 min. The enzyme was stopped by the addition of 0.5 mL 200 mM sucrose-supplemented medium and the cells were collected in 1.5 mL Eppendorf tubes. The cells were centrifuged at 22×*g* and washed twice with ice-cold 200 mM sucrose-supplemented PBS. After the last wash, the supernatant were removed and the hepatocytes were lysed by the addition of 200 μL distilled water followed by two freeze-thaw cycles.

### Measurement of trehalose and total (n)-O-Ac-Tre contents

The cell lysates were analyzed for protein content using a Bradford Commassie assay for cell count approximation. Trehalose content of cell lysates were measured using a trehalose assay kit following the manufacturer’s protocol. In brief, initial and final glucose contents of the samples were assayed spectrophotometrically before and after incubation of the samples with the enzyme trehalase which specifically hydrolyzes trehalose to 2 glucose molecules. For the measurement of total acetylated-trehalose variants in the cell lysate, 20 μL of cell lysate was mixed with 20 μL of 1 N sodium hydroxide (NaOH) and incubated over night at 70°C to fully hydrolyze the acetylated-trehalose to trehalose. Then, 20 μL of 1 N hydrochloric acid (HCl) was added to the mixture to neutralize the excess NaOH. This solution was analyzed for total trehalose content against the trehalose controls prepared in a similar fashion, and the concentration was adjusted for the dilution due to addition of NaOH and HCl. To estimate the number of cells, protein content of the cell lysate was assessed using the Pierce^TM^ Coomassie Plus (Bradford) assay (Life Technologies, Grand Island, NY, USA). In brief, 250 μl of the Coomassie Plus reagent was added to 5 μl of the cell lysate in a 96-well plate and the absorbance value was obtained after 15 min in a VersaMax microplate reader (Molecular Devices, Sunnyvale, CA, USA). Calibration of hepatocyte protein content were performed for the cells obtained from each animal by lysing and measuring the protein content of a known number of cells calculated using a hemocytometer. The obtained cell number was used to calculate total cell volume to calculate the concentrations. Cell volume measurement were performed on freshly isolated hepatocytes using a Coulter Counter Z1 cell size analyzer (Beckman Coulter, Brea, CA, USA) and 30% osmotically-inactive cell volume fraction was assumed.

### Cell viability assay

To evaluate cell viability after incubation with acetylated trehalose compared to control group, hepatocytes were seeded on fibronectin-coated plates and incubated for 24–48 h before incubation with 30 mM 6-O-Ac-Tre for 7 h, after which the medium was replaced with MTT reagent diluted 1:10 in medium to 0.5 mg/ml. The cells were incubated with the MTT reagent for 2 hrs. At the end of incubation, the MTT reagent was removed and the cells were incubated with DMSO for 10 min to dissolve all crystallized formazan. Then, the concentration of formazan was assayed by reading the absorption values of the DMSO solution in a spectrophotometric plate reader at 560 nm.

### Hepatocyte albumin assay

To determine whether acetylated trehalose loading and the presence of intracellular trehalose had an effect on cell function, we measured the albumin production up to 14 days post-loading. The hepatocytes were seeded at 0.6×10^6^ cell/well on the collagen gel in 12-well plates. Collagen gel was formed by the addition of 200 μL of collagen solution to each well and incubation for 20 min at 37°C. The cells were allowed to attach and spread at least 24 h before the experiments. For loading, hepatocytes were incubated with 30 mM 6-O-Ac-Tre in medium for 7 h, after which the medium was removed and cells were coated with 200 μL of diluted collagen solution. After the collagen solution solidified, 0.5 mL of medium was added per well and the plate was incubated at 10% CO_2_ and 37°C. Every 24 h for 14 days, the medium from each well was separately collected and 0.5 mL fresh medium was added to each well. The collected samples were stored at -80°C freezer until analyzed.

Albumin secretion was assayed following an ELISA protocol developed in-house. Briefly, 96-well high-binding ELISA plates were coated with 5 μg/well rat albumin in 100 μL PBS and incubated overnight at 4 ºC. Coated plates were washed with PBS-Tween (0.05%) at least 3 times and 50 μL of the media or standards were added to wells in the plate. Albumin antibody was diluted 1:10000 in PBS-tween and 50 μL was added to each well and incubated overnight at 4 ºC. At the end of incubation, the plates were washed with PBS-0.05% Tween at least 3 times. A substrate solution of o-phenylenediamine dihydro-chloride (OPD, 400 μg/mL) and 4 μM H_2_O_2_ was prepared in a citric acid buffer. 50 μL of the solution was added to each well and incubated for 5 min. Reaction was stopped by addition of 50 μL 8N sulfuric acid and the difference in absorbance was measured at 490 nm.

### Mitochondrial function and imaging

To determine mitochondrial function after incubation with acetylated trehalose, we used JC-1 for membrane potential and Mito-Tracker Red CMRox for imaging following the manufacturers’ protocols. The hepatocytes were seeded in fibronectin-coated plates and were allowed to attach and spread for 24–48 h. Then, the cells were incubated with 30 mM 6-O-Ac-Tre and 25 mM HEPES for 6 h, after which the wells were washed with excess medium and the cells incubated for 24 h before the assay. Immediately upon the start of incubation with staining agents, the plates were placed in the fluorescent plate reader and the fluorescence was read for 30 min at 579/599 nm excitation/emission wavelengths for Mito-Tracker and at 485 nm excitation and 530/590 nm emission wavelengths for the green and red channels respectively of JC-1. For imaging mitochondria, the hepatocytes were cultured on collagen-coated glass cover slips for 48 h, and were stained with Mito-Tracker Red 24 h after the incubation with 6-O-Ac-Tre for 6 h. The control group was prepared without incubation with 6-O-Ac-Tre. The cover slips were then washed with excess PBS and imaged using a Zeiss microscope with fluorescence capability.

### Diffusion-reaction model

**Fig 2:**
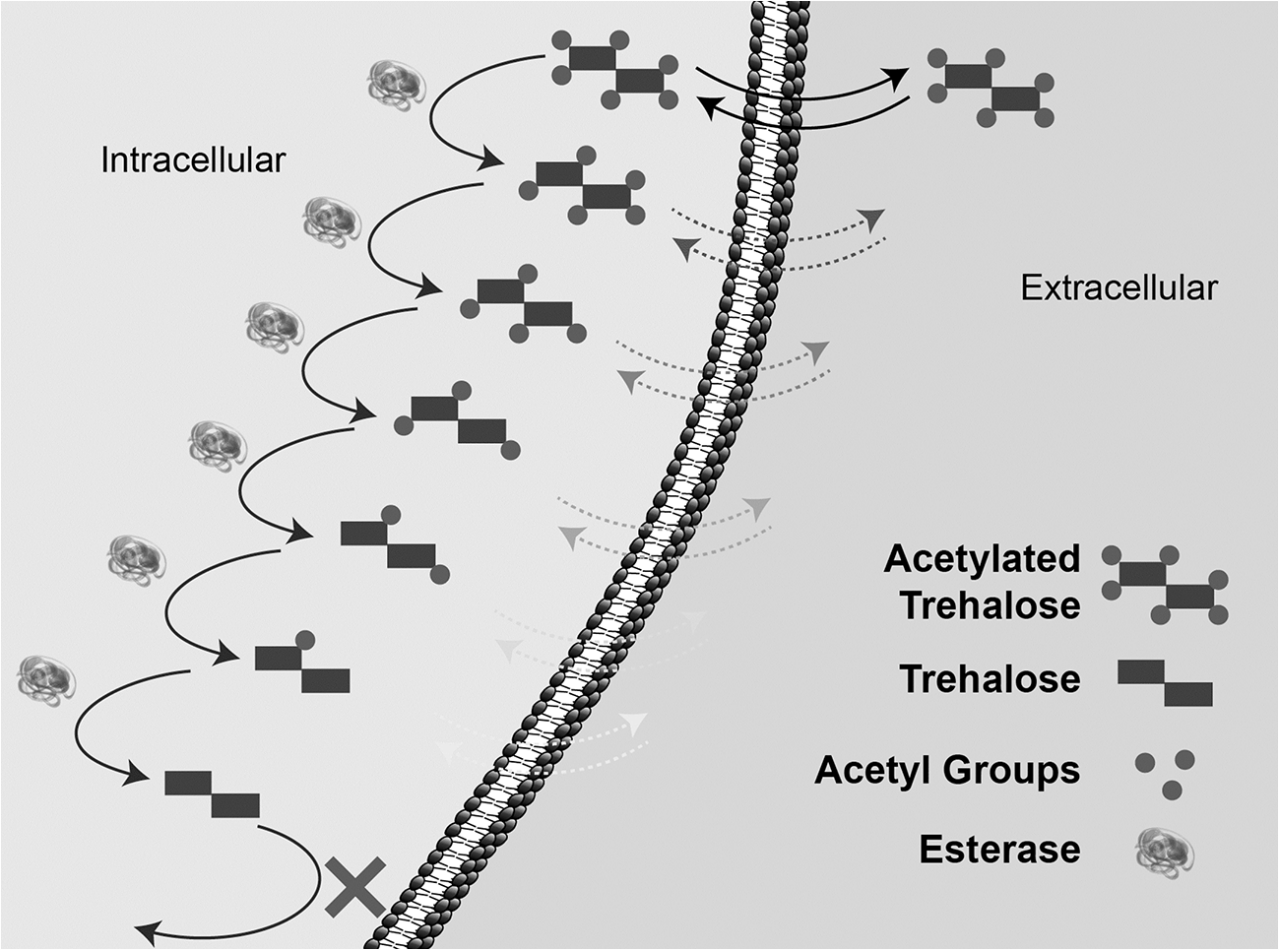
Schematic of a proposed diffusion-reaction model for de-acetylation of 6-Ac-Tre in cells.

We assume a lumped passive diffusion model for (n)-O-Ac-Tre across the cell membrane, the rate of change in the intracellular concentration of variants of (n)-O-Ac-Tre, *C*_*n*_, may be expressed using Fick’s first law:

$$\frac{d\left[C_{n}\right]}{dt}=-\frac{P_{n}A}{V}\left(\left[C_{n}^{*}]-[C_{n}\right]\right),$$ (1)

where *P*_*n*_ is the membrane permeability of (n)-O-Ac-Tre. Parameters A and V are cell surface area available for transport and cell volume, respectively. $C_{n}^{*}$ is the extracellular concentration of (n)-O-Ac-Tre, which is equal to zero for all n<6. For the intracellular de-acetylation of (n)-O-Ac-Tre to (n-1)-O-Ac-Tre by esterase activity, we assume enzymatic reaction kinetics as the following:

$$\left(\text{n}\right)\text{-O-Ac-Tre}+\text{E}\;\begin{array}{c} \overset{k_{n}^{f}}{\to }\\ \underset{k_{n}^{r}}{\leftarrow } \end{array}\;\left[\left(\text{n}\right)\text{-O-Ac-Tre}+\text{E}\right]\;\overset{k_{n}^{cat}}{\to }\left(\text{n-}1\right)\text{-O-Ac-Tre}+\text{E}$$

where $k_{n}^{f},\;k_{n}^{r}$ and $k_{n}^{cat}$ are the kinetic reaction constants for (n)-O-Ac-Tre variants ranging from 6 to 0. We may assume that the process of de-acetylation of 6-O-Ac-Tre is a series of parallel first order enzymatic reactions competing for enzyme molecules (Fig 2).

Based on the proposed reaction model, the rate of change in the concentration of (n)-O-Ac-Tre ([*C*_*n*_]) and the intermediate ([*C*_*n*_ *E*]) can be expressed as:

$$\frac{d[C_{n}]}{dt}=-k_{n}^{f}\left[C_{n}\right]\left[E_{n}\right]+k_{n}^{r}\left[C_{n}E\right]+k_{n+1}^{cat}\left[C_{n+1}E\right]-\frac{P_{n}A}{V}\left([C_{n}]-[C_{n}^{*}]\right)$$ (2)

$$\frac{d[C_{n}E]}{dt}=k_{n}^{f}\left[C_{n}\right]\left[E_{n}\right]-k_{n}^{r}\left[C_{n}E\right]-k_{n}^{cat}\left[C_{n}E\right]$$ (3)

In the above set of equations, [*E*_*n*_] is the fraction of the free enzyme that would interact with [*C*_*n*_]. Assuming quasi-steady-state condition, the left-hand-side of Eq (3) is zero. Hence, the concentration of the intermediate [*C*_*n*_ *E*] can be determined:]

$$\left[C_{n}E\right]=\frac{k_{n}^{f}\left[C_{n}\right]\left[E_{n}\right]}{\left(\;k_{n}^{r}+k_{n}^{cat}\right)}$$ (4)

We may assume that the probablity of the free enzyme interaction with variants of 6-O-Ac-Tre is directly proportional to respective mole fractions, *x*_*n*_:

$$\left[E_{n}\right]=\left[E\right]\frac{\left[C_{n}\right]}{\sum_{n}\left[C_{n}\right]}=\left[E\right]\;x_{n}$$ (5)

where [*E*] is the concentration of the free enzyme, which can also be expressed as [*E*] = [*E*_0_]−∑[*C*_*n*_ *E*]. By replacing Eq (5) in Eq (4), summation over *n*, and rearranging, we get:

$$\left[E\right]=\frac{\left[E_{0}\right]}{1+\sum \left(\frac{\left[C_{n}\right]\left[x_{n}\right]}{K_{n}^{m}}\right)}$$ (6)

where $K_{n}^{m}=\frac{k_{n}^{r}+k_{n}^{cat}}{k_{n}^{f}}$ is equivalent of the Michaelis-Menten constant for the enzymatic break-down of (n)-O-Ac-Tre variant. After substituting Eq (6) and Eq (4) in Eq (2) and rearranging, we can propose the rate of change in the concentration of (n)-O-Ac-Tre as the following:

$$\frac{d[C_{n}]}{dt}=-\frac{V_{n}^{max}\left[C_{n}\right]x_{n}}{K_{n}^{m}\left(1+\sum \left(\frac{\left[C_{n}\right]\left[x_{n}\right]}{K_{n}^{m}}\right)\right)}+\frac{V_{n+1}^{max}\left[C_{n+1}\right]x_{n+1}}{K_{n+1}^{m}\left(1+\sum \left(\frac{\left[C_{n}\right]\left[x_{n}\right]}{K_{n}^{m}}\right)\right)}+\frac{P_{n}A}{V}\left(\left[C_{n}^{*}]-[C_{n}\right]\right)$$ (7)

where $V_{n}^{max}=k_{n}^{cat}\left[E_{0}\right]$. For trehalose and 6-O-Ac-Tre, the first and second terms on the rights hand side of Eq (5) are omitted, respectively. Eq (7) is the general form of the Michaelis-Menten formulation of a multi-step enzymatic reaction for this specific case.

The permeability coefficient *P*_*n*_, and the coefficients $V_{n}^{max}$ and *K*_*n*_ in the above list of equations are unknown since no data is available in the literature on these novel molecules to be used in the model. Therefore, the values of these parameters were obtained by fitting the model to the data using a custom-written code in MATLAB (The MathWorks, Natick, MA, USA).

## Results

### Accumulation of trehalose and acetylated trehalose in cells

**Fig 3:**
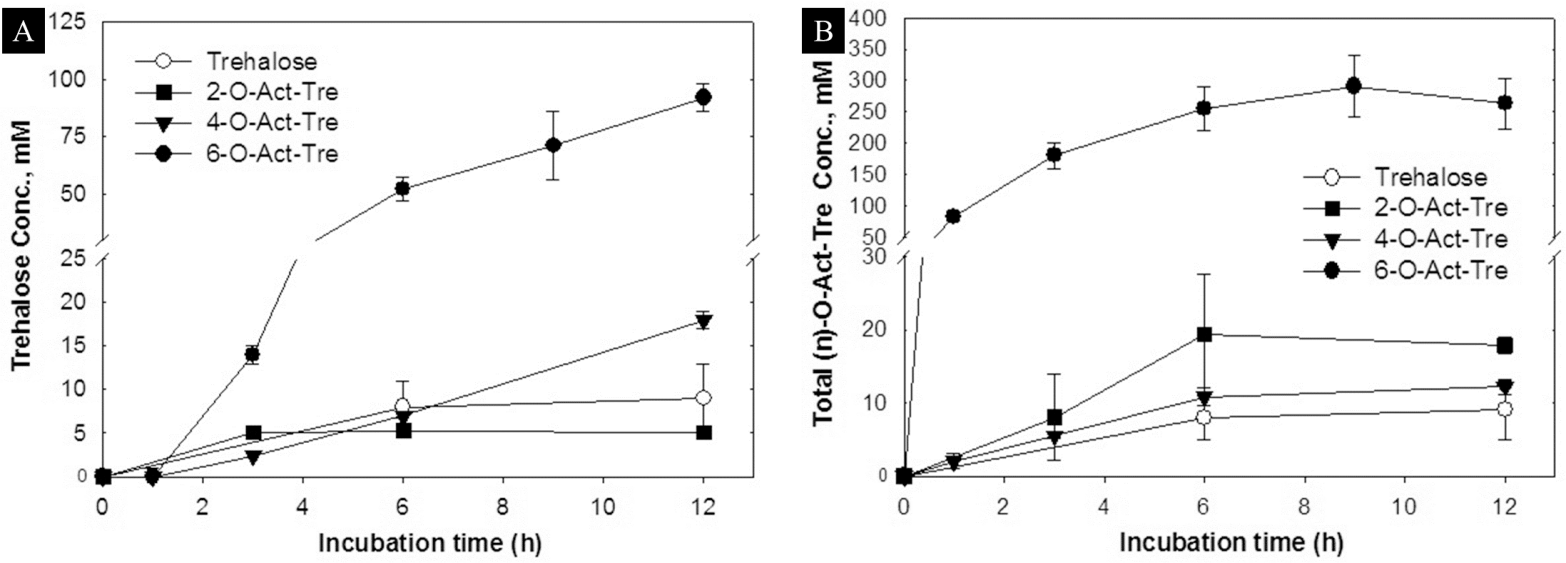
Uptake of acetylated trehalose in hepatocytes. (A) Intracellular accumulation of trehalose, and (B) intracellular accumulation of (n)-O-Ac-Tre, after incubation with 30 mM concentration of trehalose, 2-, 4-, and 6-O-Ac-Tre. 8-O-Ac-Tre was not studied as it was not water soluble. Error bars represent SD (n = 3). (* denote p>0.05 and # denote p<0.05 between respective groups).

We hypothesized that acetylation of trehalose would increase its membrane permeability, and that once in the cytoplasm, endogenous esterases would hydrolyze acetylated-trehalose to trehalose. To test this hypothesis, we measured the concentration of non-acetylated trehalose in rat hepatocytes after incubation with 30 mM solutions of trehalose and 2-, 4- and 6-O-Ac-Tre at regular intervals (Fig 3A). To reduce hydrolysis of acetylated trehalose by esterase activity after lysis, the cell lysate were immediately frozen or kept on ice until analyzed. The detection and time-dependent increase of trehalose in cell lysate suggests that both the penetration and the hydrolysis of acetylated trehalose occurred. However, a significant difference was observed in the amount of converted trehalose when comparing 6-O-Ac-Tre to 2- and 4-O-Ac-Tre. With 6-O-Ac-Tre, the trehalose concentration showed a time-dependent increase, suggesting continuous permeation and hydrolysis of 6-O-Ac-Tre. In the first hour of incubation, the concentration of fully deacetylated trehalose was below the detection limits of our trehalose assay (<1 mM), but increased linearly between 3–12 h of incubation with an approximate rate of 8.5 mM/h. After 12 h, the intracellularly-accumulated trehalose reached as high as 92 mM. This is more than 3 fold higher than the incubation concentration for 6-O-Ac-Tre. The amounts of trehalose accumulated in cells after incubation with 2- and 4-O-Ac-Tre were much smaller. Between 3–12 h, the trehalose concentration reached a plateau at ~8 mM in cells incubated with 2-O-Ac-Tre, while it gradually but slowly increased to ~17 mM in cells incubated with 4-O-Ac-Tre.

The full hydrolysis of each acetylated trehalose molecule may not occur instantaneously but gradually, producing (n)-O-Ac-Tre molecules with 0≤n<6. Hence, a mixture of incompletely deacetylated forms of trehalose may be present within the cell at all times. To fully characterize the permeability and uptake of trehalose by acetylation, we also measured the total concentration of (n)-O-Ac-Tre in cells (Fig 3B). For 6-O-Ac-Tre, a time-dependent increase in the concentration of (n)-O-Ac-Tre was observed, reaching a maximum of ~300 mM after 9 h and appeared to reach a plateau by 12 h. For 2- and 4-O-Ac-Tre, the intracellular accumulation of (n)-O-Ac-Tre did not significantly change over time, plateauing at 20 mM and 14 mM, respectively. The uptake of trehalose, 2-Act-Tre and 4-Act-Tre are in similar range, while 6-Act-Tre uptake is at least one order of magnitude higher. Hence, while endocytosis might play role in the uptake of all variants of trehalose in this study, another mechanism, most likely diffusion, is responsible for higher uptake of 6-Act-Tre in cells. This suggests that diffusion is most likely the mechanism for intracellular delivery of 6-Act-Tre.

A novel observation was the accumulation of acetylated trehalose derivatives in cells to higher than the incubation concentration. When cells were incubated with 30 mM 6-O-Ac-Tre, the intracellular concentration reached ~250–300 mM total (n)-O-Ac-Tre, which represents ~8–10 fold the extracellular concentration. Such an accumulation phenomenon was not observed with 2- and 4-O-Ac-Tre. In this regard, 6-O-Ac-Tre was both effective and efficient for intracellular trehalose delivery. Although 2- and 4-O-Ac-Tre are more lipophilic than regular trehalose, we did not observe a significant enhancement of the permeability for the specific arrangement of the acetyl groups in these molecules in this study. These results were aligned with our hypothesis on increased membrane permeability of trehalose by conjugation to lipophilic groups. The results presented in Fig 3 demonstrate that the increased lipophilicity of the molecules greatly enhanced the membrane permeability and intracellular accumulation. However, no direct relationship between the number of acetyl groups and increased permeability was observed.

### The diffusion-reaction model for 6-O-Ac-Tre permeation

**Fig 4:**
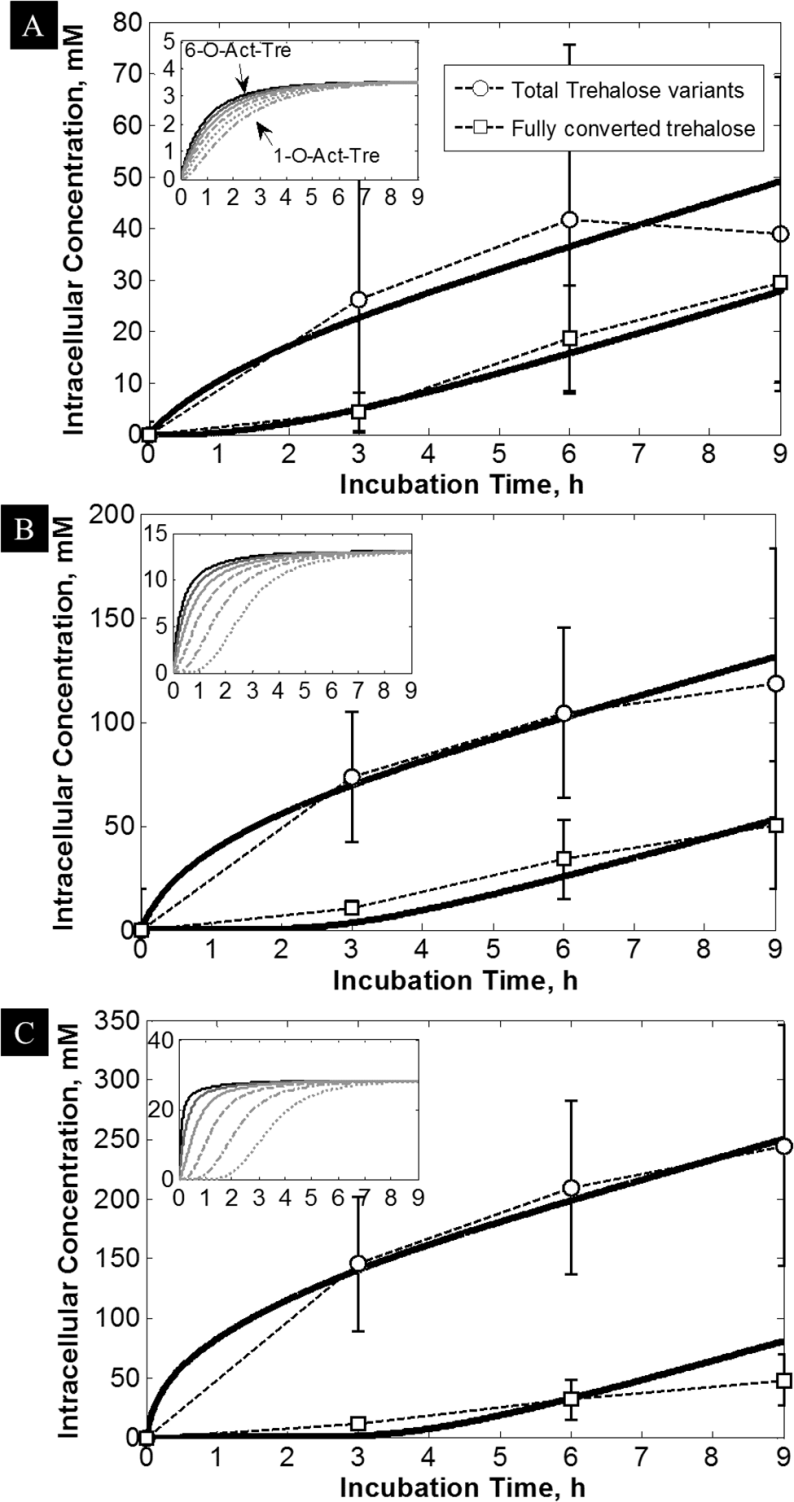
Fitting results of the proposed diffusion-reaction model to the data. With 3 fitting parameters, the model (solid lines) closely followed the averaged uptake data for trehalose (squares) and total (n)-O-Ac-Tre (circles), which were obtained by incubation with (A) 5mM, (B) 15 mM, and (C) 30 mM 6-O-Ac-Tre. Inset Figures: Predicted increase in the concentration of 6- (black solid line), 5- (dark-gray solid line), 4- (gray solid line), 3- (gray dashed line), 2- (gray dash-dotted line) and 1-O-Ac-Tre (gray dotted line). These results were obtained by averaging the data from at least 3 animals and the error bars represent the animal-to-animal variability.

The data on accumulation of trehalose and acetylated trehalose in hepatocytes were obtained using cells isolated from at least 3 different animals, and demonstrated a similar trend. However, parameters such as size/age/health and variability in the isolation procedure on different days could potentially affect the function of the hepatocytes from different animals. For the purpose of calculating average values for diffusion and conversion of Act-Tre, the average values are plotted in one figure (Fig 4) where the large error bars represent the data range for visual evaluation of the goodness of the fit. The individual data sets are reported in S1 Fig.

Our best results for permeability and accumulation of trehalose were observed with 6-O-Ac-Tre. The proposed diffusion-reaction model in the Methods section allows calculating the permeability coefficients for 6-O-Ac-Tre by fitting the model to the experimental results for 6-O-Ac-Tre. First, we made a few simplifying assumptions. Based on the data on permeability and accumulation of 2- and 4-O-Ac-Tre presented in Fig 3, we assumed that deacetylation of 6-O-Ac-Tre would significantly reduce the membrane permeability of the remaining conjugate. Hence, we assumed that the permeability of (n)-O-Ac-Tre (*P*_*n*_, n<6) would be negligible compared to that of the parent molecule. We also assumed that the enzymatic kinetics of the de-acetylation process were similar for all the steps of hydrolysis of (n)-O-Ac-Tre to (n-1)-O-Ac-Tre. Therefore, $K_{n}^{m}=K^{m}$ and $V_{n}^{max}=V^{max}$ for n≤6. Hence, the model was fit to data using 3 parameters, i.e. *K* ^*m*^, *V* ^*max*^ and *P*.

The model was fit to 3 separate data sets obtained from incubation of cells with 5, 15 and 30 mM 6-O-Ac-Tre, each obtained consisting out of total trehalose and total (n)-O-Ac-Tre variants concentration data (Fig 4, also see S1 Fig). The model was fit to the data up to 9 h incubation. The results are plotted in Fig 4. Using only 3 fitting parameters, the model fit all the 6 data sets very closely with nonlinear regression coefficient R^2^ values >0.92. The fit values for *K* ^*m*^, *V* ^*max*^ and *P* are reported in Table 1. By increasing the incubation concentration from 5 to 30 mM, the value of permeability increased from 2.2×10^−9^ to 8.0×10^−9^ m s^-1^ resulting in an average of 5.0±3.4×10^−9^ m s^-1^. The value of *V*_*max*_ also increased from 0.007 to 0.027 mM s^-1^ while *K*_*m*_ remained constant (~0.1 mM). Per definition, *K*_*m*_ is the substrate concentration (here (n)-O-Ac-Tre) at which the reaction rate reaches half of the *V*_*max*_, and a very small value for *K*_*m*_ suggests a high affinity between the substrate and the enzyme.

**Table 1:** Best fit model parameters.

C* (mM)	P (m s^-1^)	*K* ^m^ (mM)	*V* ^*max*^ (mM s^-1^)	*R* ^*2*^
5	2.2×10^−9^	0.09	0.007	0.922
15	3.9×10^−9^	0.1	0.016	0.983
30	8.0×10^−9^	0.1	0.027	0.981
Average±S.D.	5.0±3.4×10^−9^	0.097±0.006	0.0167±0.01	

The insets in Fig 4A, 4B and 4C show the predicted time-dependent increase in the concentration of (n)-O-Ac-Tre variants within the cell. The predicted concentrations of (n)-O-Ac-Tre at steady state (i.e. after 3 h) plateaued toward values close to but less than the extracellular concentration of 6-O-Ac-Tre. Increasing the extracellular incubation concentration caused an intracellular crowding effect which delayed the appearance of fully hydrolyzed trehalose (Fig 4C). The predictions show that, at 5 mM incubation concentration, 1-O-Ac-Tre (indicating rapid hydrolysis) appeared almost instantaneously, while for 15 and 30 mM, this time increased to >1 h (indicating a backlog effect in hydrolysis).

### Effect of trehalose loading on cell viability and function

**Fig 5:**
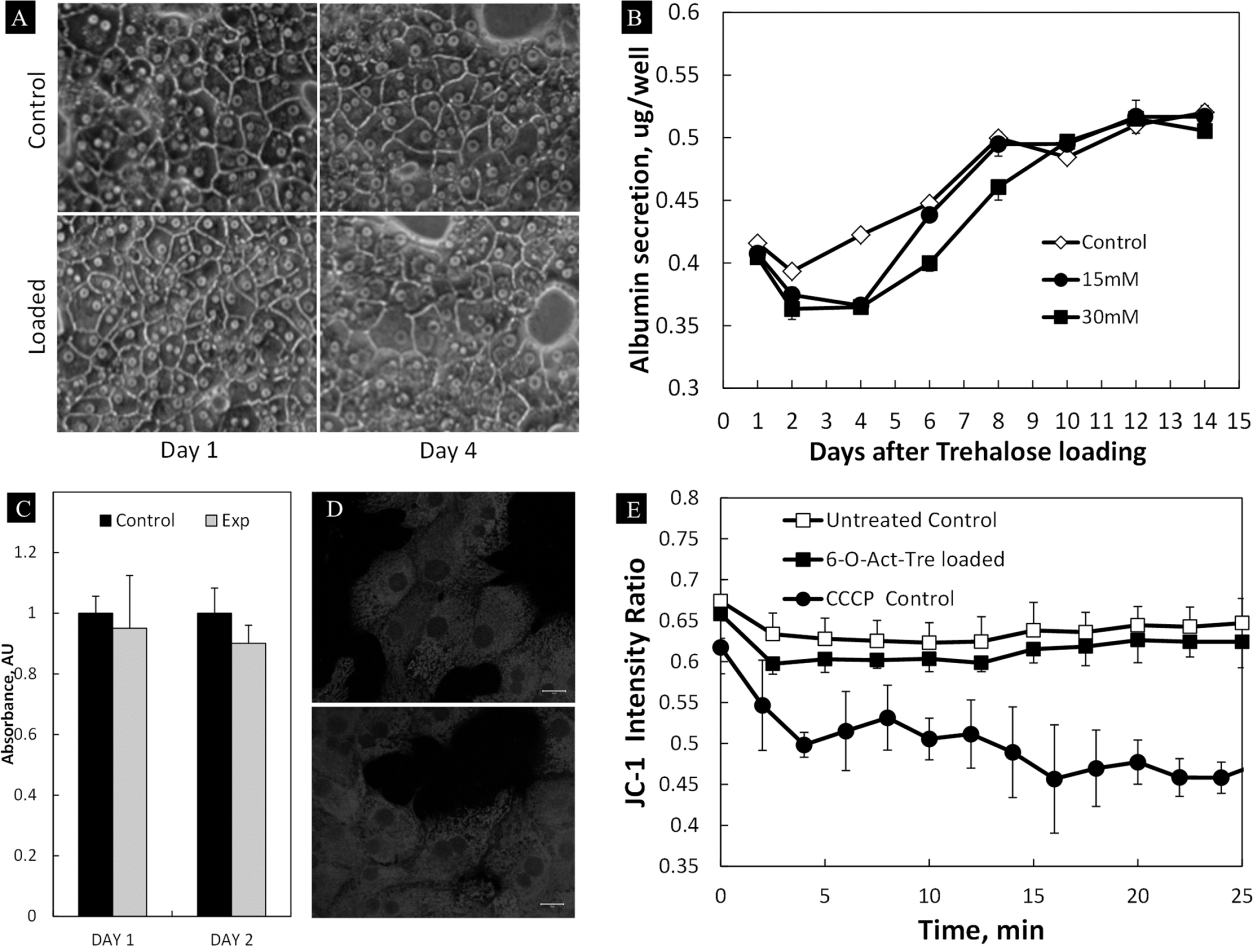
Assessment of hepatocyte functional viability post-incubation with acetylated trehalose. (A) Overall morphology 1 and 4 days after incubation with 30 mM 6-O-Ac-Tre (scale bar = 20 μm), and (B) a 14-day follow-up of metabolism in rat hepatocytes by measuring daily albumin secretion showed a slow recovery of metabolism in trehalose-loaded cells compared to the control group. (C) The MTT viability assay showed no significant difference in the viabilities of the experimental and the control groups 48 h after trehalose loading with 30 mM 6-O-Ac-Tre (p>0.05). (D) Confocal imaging of mitochondria stained using Mito-Tracker Red 12 h after incubation with 6-O-Ac-Tre showed the presence of mitochondria competent to accumulate dye (dependent on mitochondrial membrane potential, ΔΨ) in both the control (top) and the experiment (bottom) groups. Scale bar = 10 μm (E) JC-1 staining after 6 h incubation with 6-O-Ac-Tre indicates similar mitochondrial ΔΨ in experimental and control groups. A positive control group incubated with the uncoupler CCCP during staining is shown for comparison. Error bars represent SD (n = 3).

We next examined the long-term functional activity of hepatocytes after incubation with 6-O-Ac-Tre. The cells were incubated for 14 days following the incubation with 15 and 30 mM 6-O-Ac-Tre for 6 h (Fig 5). During the post-loading incubation, the cells were examined for overall morphology, viability and the albumin secretion. Mitochondrial membrane potential (ΔΨ) was also examined. Qualitatively, minimal changes in overall morphology and lipid droplet formation, both indicators of stress, were observed in the experimental group compared to the control group. Distinct cell boundaries and clear nuclei were maintained, suggesting live and functional cells (Fig 5A). Albumin secretion showed a slow-down in cell function in the experimental group within 3–4 days post-loading (Fig 5B). The albumin secretion eventually increased to similar levels as the control group within 10 days after loading with 6-O-Ac-Tre. Viability of the cells as assayed by MTT crystallization during the period of low albumin secretion showed similar activity levels in both groups, suggesting no cell loss up to 48 h after loading (Fig 5C). Evaluation of mitochondrial ΔΨ using Mito-Tracker Red staining (Fig 5D) and JC-1 fluorescence intensity ratio (Fig 5E) suggested no significant difference between the treated groups and the controls in ability to maintain ΔΨ. These results suggest that the permeation and hydrolysis of 6-O-Ac-Tre and intracellular presence of trehalose did not irreversibly damage the hepatic cells in a 14-day period post-incubation.

## Discussion

Trehalose is a non-charged and hydrophilic disaccharide which does not freely penetrate the mammalian cell membrane without external interventions. These interventions expose the cell to a range of deleterious conditions, from temperature and osmotic challenges [21–23] to perforation of the membrane [29,34,27] and genetic interventions [15,28], and often lead to appreciable cell loss during trehalose loading. Genetic intervention is also prohibited in many circumstances including when the cells need be transfused or implanted in patients.

In this study, we investigated an approach to increasing the membrane permeability of trehalose by conjugation with 2, 4 and 6 acetyl groups (conjugation with 8 acetyl groups resulted in complete loss of aqueous solubility and it was not further studied). We found that conjugation with 2 and 4 acetyl groups did not significantly enhanced the membrane permeability of trehalose (Fig 3). The amounts of permeated 2- and 4-O-Ac-Tre in cells were in the same range as the amount of regular trehalose taken up by endocytosis (Fig 3). It is plausible that homologues of 2- and 4-O-Ac-Tre molecules with different spatial arrangements of acetyl groups may display different permeabilities. Interestingly, increasing the number of acetyl groups to 6 resulted in a significant improvement in trehalose permeability. In addition, we found that the hydrolysis of the permeated acetylated trehalose by endogenous intracellular esterases created a concentration gradient across the membrane resulting in continual permeation and accumulation of acetylated and hydrolyzed trehalose within the cell (Fig 3). The permeability coefficient calculated for 6-O-Ac-Tre was similar to other molecules known to easily permeate cell membrane [35], and the reaction kinetics of the hydrolysis of acetylated trehalose suggested an average conversion rate of 60±36 mM h^-1^ by esterases (Fig 4). The accumulation of trehalose and its acetylated variants in rat hepatocytes reached as high as 250±49 mM within 6–9 h of incubation, beyond which it plateaued. Interestingly, such high amounts of intracellular trehalose didn’t appear to adversely impact the viability and function of the hepatocytes. Although the presence of intracellular trehalose is expected to increase intracellular osmolality and induce osmotic swelling, several cell types have been observed to tolerate the osmotic impact of various levels of intracellular trehalose [26,29,36,37]. A byproduct of 6-O-Ac-Tre hydrolysis is acetic acid which can result in a significant reduction in intracellular pH. Nonetheless, we found that the release of acetic acid had minimal impact on the functional viability and long-term albumin secretion in rat hepatocytes (Fig 5). This approach to trehalose loading only manipulates the chemical properties of trehalose, and not the cell, to increase membrane permeability to trehalose.

The rate of trehalose loading using 6-O-Ac-Tre was generally slower than most other techniques that involve poration of the membrane [29,34], but is comparable to fluid phase endocytosis in platelets [30] and engineered TRET1 transporter protein [28,38]. In membrane poration techniques, the yield of trehalose delivery can be increased by elongation of the pore opening time which consequently increases cell death. Therefore, cells are incubated with a high concentration of trehalose and the pore opening time is limited to a few minutes to maximize the yield of delivery against cell loss. A notable advantage of 6-O-Ac-Tre was that only a small extracellular concentration (30 mM) was sufficient for continuous increase and accumulation of trehalose+acetylated variants in cells 7–10 times higher than the extracellular concentration (Fig 3); non-acetylated trehalose was accumulated intracellularly three-fold higher (i.e., 92 mM) compared to the external concentration of 6-O-Ac-Tre. This was consistently observed in all the cell types that we examined (S2 Fig), and we achieved as high as 1000% yield of delivery for trehalose and its acetylated variants in some cell types after 6–9 h of incubation. The potential of acetylated trehalose variants to offer protection against freezing and drying is currently under study. Longer incubation periods after the initial loading of 6-O-Ac-Tre will allow full hydrolysis of all acetylated trehalose variants to trehalose. Compared to other techniques, such efficiency for trehalose delivery is unprecedented. The calculated permeability coefficient suggests that the permeation of 6-O-Ac-Tre is relatively fast, and the limiting factor for the uptake of trehalose is the rate of hydrolysis of acetylated trehalose by endogenous esterases (Fig 4C and inset). The hydrolysis of 6-O-Ac-Tre creates a concentration gradient across the cell membrane resulting in constant influx of 6-O-Ac-Tre. Since the amount of intracellular esterases is limited, this creates a backlog effect resulting in slow down of the turnover rate for trehalose. Removal of the external supply of 6-O-Ac-Tre after an initial loading period would help reducing the backlog and increasing the turnover rate of trehalose (S3 Fig). This can be used as a strategy to increase trehalose turnover and delivery rates in cells.

Engineering trehalose chemical structure is an innocuous, cell-friendly method for intracellular trehalose delivery which can overcome a major obstacle in biopreservation studies using trehalose. Acetylation is only one way of increasing the lipophilicity and consequent membrane permeability of trehalose. In a similar fashion, the application of a range of lipophilic molecules can be studied and optimized for the same purpose. This approach provides an exciting opportunity for investigating the trehalose potency as an intracellular, nontoxic protective agent in various cell types. The biopreservation studies using this approach are in progress.

## Supporting Information

S1 Fig Time- and concentration-dependent increase in trehalose (frames A, C and E) and total (n)-O-Ac-Tre (frames B, D and F) concentration after incubation with 5, 15 and 30 mM 6-O-Ac-Tre in cells isolated from 3 different animals. (TIFF)

S2 Fig Uptake of 6-O-Ac-Tre and conversion to Trehalose in various cells (human embryonic kidney cell line HEK 293, normal human dermal fibroblasts, human hepatoma cell line HepG2-C3A, and rat hepatocytes) after incubation with 15 mM 6-O-Ac-Tre for 12 h. The uptake of trehalose after incubation with same concentration of regular trehalose is also provided for NHDF and rat hepatocytes.
(TIFF)

S3 Fig Trehalose and total (n)-O-Ac-Tre concentration change after removal of extracellular gradient of 6-O-Ac-Tre. Total (n)-O-Ac-Tre concentration plateaus at approximately 105 mM after 8h, confirming that (n)-O-Ac-Tre (n<6) is trapped within the cell. Trehalose full conversion continues at a higher rate (~10 mM/h) than when the 6-O-Ac-Tre gradient is present (6.6 mM/h).
(TIFF)
